# The collapse of distance

**DOI:** 10.1093/oncolo/oyag306

**Published:** 2026-07-31

**Authors:** Jina Bai

**Affiliations:** Department of General Internal Medicine, Section of Hospital Medicine, New York Presbyterian-Weill Cornell Medical Center, New York, NY 10065, United States

My husband was 34 when he was diagnosed with metastatic hepatocellular carcinoma. We had just returned from a two-week trip to New Zealand—hiking the Tongariro Alpine Crossing, driving across the North and South Islands. He had never been ill before. He rarely saw physicians.

My life was unfolding as I had once imagined—building a career in a big city, working as a physician assistant caring for patients with advanced illness, and starting a family. We were in the early years of our marriage, having recently bought a small apartment in Queens and building what felt like a predictable future.

The first sign was jaundice. I remember the moment I noticed it—subtle at first, something I almost dismissed. Then came the ultrasound report and the feeling that something irreversible had already begun, even before we had the name for it. Within days, we moved through CT scans, MRIs, endoscopy—each test solidifying the reality I was not ready to face. When the metastatic disease was finally confirmed, my mind went quiet. Not denial exactly, but a blankness. I knew what it meant.

In the weeks that followed, time lost its usual structure. Appointments blurred into one another. Hospital visits became routine. Chemotherapy, infection, GI bleed, hospitalization, discharge—repeat. What I recognized in my professional life—systems, protocols, prognosis—now belonged to someone I loved, and the familiarity offered no comfort.

People often speak of the stages of grief. I experienced them not as stages, but as cycles that repeated without warning. I would wake up in the morning in shock, as if I had forgotten overnight what had already happened. At other times, guilt overwhelmed me: *How did I not see this earlier? How did I miss it in someone I have known so closely?* Even knowing, rationally, that hepatocellular carcinoma does not announce itself in advance, I could not fully separate knowledge from self-blame.

As my world was shattering, I needed to talk to people. I went to a caregiver support group, but I did not fit in. When others spoke of ungrounded hopes, I often left feeling more unsettled. I found myself wishing for someone like me—someone experiencing a loved one’s illness through the lens of a clinician.

There is an unspoken but persistent assumption among clinicians that proximity to illness confers a kind of protection—that knowing more makes one less vulnerable. I had seen illness hundreds of times in patients. I had explained diagnoses, prognoses, and trajectories. Yet none of that prepared me for the collapse of distance between being a clinician and a family member when the patient was my husband. Illness was no longer something I observed. It was something I lived with.

During one of his many hospitalizations, I tried to ask about his code status preferences, but I couldn’t bring myself to do it. His attending physician pulled me aside in the hallway and said, “You don’t have to be his PA. Just be his wife.” Later that day, he told me my husband had chosen DNR/DNI. I felt a measure of relief—until then, I had not known whether my husband truly understood the prognosis, and this suggested that he did. At the same time, that clarity carried a deep sadness.

During the two years of his illness, work became my only stable ground. In the hospital, I could function. I could focus on tasks, decisions, and routines that required clarity. The moment I stepped away from work, I returned to a different reality—one where I was no longer a clinician but a wife watching her husband decline. Work did not cure anything, but it allowed me to temporarily set grief aside.

He never spoke about fear. Near the end of his life, he told me he had no regrets in life. Then, holding my hand, he said, “But I messed up yours.” I remember the steadiness in his voice, the absence of self-pity. I did not know how to respond. It still amazes me that a 35-year-old could face death with such calm.

He died at 36.

In the years since he left me, I have continued to work in medicine. Time has not made grief disappear, but it has changed its shape. I still miss him. I continue to live fully, as he did. The memory of his illness and death has softened into the larger memory of our life together, held alongside the joy we shared.

Writing this feels like a threshold I have not previously been able to cross. I have kept this story private while continuing to care for patients in suffering. Perhaps this is the first time I am allowing the two versions of myself—the clinician and the wife—to occupy the same space on the page.

What I carry forward is not a lesson so much as a reorientation. Illness does not belong to others. It is not distant from the clinician, nor softened by familiarity with medical language. It arrives without regard for knowledge, profession, or preparedness. I now bring this experience into my care of patients and their families—with the belief that even in illness, there can be steadiness, meaning, and a way forward.

